# Physics-Informed Neural Networks for Predicting Hydration Degree in Cementitious Systems Blended with Slag and Fly Ash

**DOI:** 10.3390/ma19142978

**Published:** 2026-07-10

**Authors:** Xiaoyi Hu, Xiaofeng Liao, Zaiyi Liao, Zhizhi Wang, Min Gan

**Affiliations:** 1 Key Laboratory of Geological Hazards on Three Gorges Reservoir Area, Ministry of Education, China Three Gorges University, Yichang 443002, China; 2 School of Urban Construction Engineering, Chongqing Technology and Business Institute, Chongqing 400085, China; 3 College of Civil Engineering & Architecture, China Three Gorges University, Yichang 443002, China; 4 Chongqing Zhuyun Science and Technology Limited Corporation, Chongqing 400045, China; 5 School of Civil Engineering, Chongqing University, Chongqing 400045, China; 6 State Key Laboratory of Safety and Resilience of Civil Engineering in Mountain Area, Chongqing University, Chongqing 400045, China

**Keywords:** physics-informed neural network, degree of hydration, cement hydration, supplementary cementitious materials, slag, fly ash

## Abstract

Accurately predicting the degree of hydration in cement systems blended with slag and fly ash is difficult when experimental data are limited. Empirical kinetic models typically need recalibration for each specific blend, while purely data-driven approaches often fail to maintain physical consistency. In this work, we present a physics-informed neural network for predicting isothermal hydration by incorporating the Avrami–Erofeev–Arrhenius ordinary differential equation into the training loss and using a composition-focused sub-network to relate blend proportions to blend-specific kinetic parameters. The model was trained and tested on 29,379 calorimetry data points from 77 multi-component cement systems gathered from two open-access datasets. For previously unseen test systems, it reached an $R^{2}$ of 0.9864 and a symmetric mean absolute percentage error (sMAPE) of 25.3%. Its overall sMAPE was lower than that of a neural network with the same architecture but without physics-based constraints (34.9%); stage-resolved analysis showed that the largest difference occurred during early hydration. Across eight random seeds, the physics constraint did not confer a data-efficiency or training-stability advantage over the same-architecture network. The distinguishing features of the PINN are instead its explicit ODE and monotonicity regularization and its composition-conditioned effective parameters. Although random forest regression produced lower pointwise error, it failed to maintain physically consistent hydration-rate behavior. Overall, the framework provides a composition-conditioned surrogate that incorporates kinetic regularization within the composition range investigated.

## 1. Introduction

The incorporation of supplementary cementitious materials (SCMs)—including ground-granulated blast-furnace slag, fly ash, calcined clay, and limestone—into ordinary Portland cement (OPC) is a key strategy in sustainable concrete production [1,2]. These materials contribute to lower ${\mathrm{CO}}_{2}$ emissions, improved long-term durability, and the beneficial reuse of industrial by-products [3,4,5,6]. At the same time, the hydration kinetics of multi-component systems are considerably more complex than those of pure OPC blends, since each SCM brings its own reaction mechanism—whether hydraulic, pozzolanic, or filler-related—with distinct rate constants, activation energies, and interactions with clinker hydration products [7,8]. The degree of hydration α(t) denotes the overall (bulk) degree of reaction of the binder, not the clinker-phase value. It is calculated as the cumulative heat released at time *t* divided by the blend’s total potential heat. Its evolution depends nonlinearly on blend composition, water-to-binder ratio, and curing temperature [9,10].

Reliable prediction of α(t) is essential for several engineering applications: (i) estimating compressive strength development through strength–degree-of-hydration relationships [11,12], (ii) calculating heat generation rates for thermal analysis of mass concrete [13,14], and (iii) optimizing blend proportions to achieve target performance. Isothermal calorimetry remains the benchmark method for measuring α(t) [15], but experimentally characterizing every possible composition is prohibitively costly. A dependable predictive model that can be trained with only a limited calibration dataset would therefore greatly speed up mix design.

### 1.1. Traditional Models

Classical hydration models—including nucleation-growth kinetics of the Avrami–Erofeev type, affinity-based formulations, and multi-parameter empirical models [7,16]—offer physically meaningful descriptions of α(t) for single-component binders, but they require separate parameter calibration for each blend. For example, a representative nucleation-growth (Avrami–Erofeev) model describes α(t) as

(1) $$\alpha \left(t\right)=\alpha_{u}\;\left[1-\exp\;\left(-\;{\left(\frac{t}{\tau }\right)}^{\;n}\right)\right]$$

where $\alpha_{u}$ denotes the ultimate degree of hydration, τ represents a characteristic time scale, and *n* is the reaction-order exponent. The exponential affinity model, which is widely used in cement-hydration and thermal-analysis simulations, describes the hydration rate as follows:

(2) $$\frac{d\alpha }{dt}=B_{1}\;\left(\frac{B_{2}}{\alpha_{\infty }}+\alpha \right)\;\left(\alpha_{\infty }-\alpha \right)\exp\left(-\eta \alpha \right)\;\cdot \;\exp\;\left(-\frac{E_{a}}{RT}\right)$$

where $B_{1}$, $B_{2}$, η, $\alpha_{\infty }$, and $E_{a}$ are five blend-specific fitted parameters. Applying these approaches to multi-component systems is still difficult, because the blending rules are mainly empirical and may not fully represent systems with strong synergistic interactions [11,17].

### 1.2. Machine Learning Approaches

Machine learning methods have shown strong interpolation performance for hydration kinetics when predictions stay within the range of the training data, but they still suffer from a key limitation: without constraints from governing equations, they cannot ensure physically valid predictions when experimental data are limited. Lapeyre et al. [18] developed random forest (RF) models for 235 multi-component systems using composition as a continuous input, representing the largest machine learning study on hydration kinetics so far. Cook et al. [19] further applied this RF-based strategy to blended cement systems and demonstrated its usefulness for practical mixture optimization based on predicted heat-evolution profiles. Han et al. [20] employed deep forest alongside segmentation guided by topological constraint theory for fly ash-containing binders, allowing for effective learning from limited datasets by using domain knowledge to reduce input dimensionality. Degefa et al. [12] used genetic programming to predict the degree of reaction of slag, fly ash, metakaolin, and silica fume in hydrated Portland cement, reporting an $R^{2}$ of 0.89. Artificial neural network methods have also been applied to predict hydration heat in various cementitious composites [21,22], while gradient boosting and random forest models have been used to estimate compressive strength from mixture proportions [23,24,25,26]. Yu and Geng [27] demonstrated that deep learning can quantify GGBS hydration from backscattered electron images with accuracy comparable to PONKCS XRD, and other deep learning work has addressed cement phase segmentation [28,29] and microstructural evolution [30].

Despite these advances, purely data-driven methods still share two key limitations: (i) they rely on large calibration datasets, which may not be available for new blend compositions, and (ii) they do not explicitly impose kinetic constraints, so their predictions may include non-monotonic α(t) curves or negative heat generation rates [31]. These limitations motivate the incorporation of physics-based regularization into the learning framework.

### 1.3. Physics-Informed Neural Networks

Physics-informed neural networks (PINNs) [32] incorporate governing differential equations directly into the training loss, providing physics-based regularization that is particularly valuable when data are scarce [31]. Theoretical convergence studies [33], well-developed open-source platforms such as DeepXDE [34], and successful applications in heat transfer [35], fluid mechanics [36,37], and solid mechanics [38,39] demonstrate that physics residuals can effectively steer neural network solutions toward physically admissible regions without requiring large calibration datasets. In cement research, Ahmad et al. [40] were the first to apply PINNs to isothermal hydration by embedding the Avrami–Erofeev ODE into the training objective, showing substantially improved accuracy over a conventional ANN for single-component OPC pastes. Lee et al. [14] employed PINNs to address the inverse problem of identifying adiabatic temperature-rise characteristics in mass concrete. Rahman and Lu [41] proposed EcoBlendNet, a PINN-based framework for optimizing SCM replacement ratios, achieving low approximation error with only 5% of the training data; however, their model was trained and validated on just three fixed blend formulations rather than across a continuous composition space.

Despite these advances, a significant gap still remains. Current PINN studies on cement hydration have focused either on single-component OPC pastes [40] or on only a limited number of fixed blend formulations [41], whereas data-driven machine learning models capable of handling multi-component systems [18,19,20] do not explicitly include kinetic constraints and may require extensive calibration data. This gap is important because multi-component blends are now the industry norm—global average SCM replacement has surpassed 30% [2]—and slag and fly ash contribute fundamentally different kinetic responses: slag undergoes a latent hydraulic reaction activated by $\mathrm{Ca}{\left(\mathrm{OH}\right)}_{2}$ [16], whereas fly ash is involved in a slower pozzolanic reaction controlled by portlandite depletion [1,10].

These considerations motivate the use of a physics-embedded PINN with a composition sub-network, which represents effective kinetic parameters as continuous functions of blend composition without per-blend calibration.

### 1.4. Objectives and Contributions

The main contributions of this paper are as follows:

1. A physics-informed neural network for multi-component hydration kinetics: We develop a PINN framework for predicting α(t) across the studied slag/fly ash composition space, validated on 77 systems compiled from public datasets spanning 0–73% SCM replacement (clinker contents from 27 to 100%), without per-blend refitting. A composition sub-network maps the blend vector to blend-specific kinetic parameters that enter the ODE residual. This structure allows one model to represent the multi-component space while explicitly penalizing deviations from the governing ODE at sampled collocation points. In contrast to prior PINN studies on cement hydration—limited to single-component OPC paste [40] or to a few fixed blend formulations [41]—the present composition-conditioned framework uses a dedicated composition sub-network to model hydration kinetics across a continuously represented slag/fly ash composition space without per-blend recalibration.
2. Performance with limited training data across the composition space: Embedding the Avrami–Erofeev ODE (Equation (4)) into the training objective yields a single model that reaches $R^{2}\approx 0.90$ with about ten training systems. Across eight random seeds, the physics-constrained model performs comparably to a same-architecture ANN in this data-efficiency analysis; we therefore do not claim a seed-stability advantage. Its distinguishing features are the explicit ODE and monotonicity regularization and the effective, composition-conditioned parameters (Contribution 3), rather than reduced variance.
3. Physical regularization and effective kinetic parameters: The PINN explicitly penalizes ODE-residual and monotonicity violations. It produces no dα/dt<0 cases on the test set. The same-architecture ANN also produces no negative rates in this test, whereas the RF baseline violates monotonicity at 13.1% of the test points. The PINN also yields effective, composition-conditioned parameters $\left({\hat{B}}_{1},\hat{n}\right)$; the apparent activation energy $E_{a}$ is held fixed at a representative literature value rather than learned. Relative to the same-architecture ANN, the PINN has a lower overall sMAPE (25.3% vs. 34.9%), while their difference in $R^{2}$ is not statistically significant. The largest stage-resolved difference occurs during the induction-to-acceleration transition.

## 2. Materials and Methods

### 2.1. PINN Framework Overview

**Figure 1:**
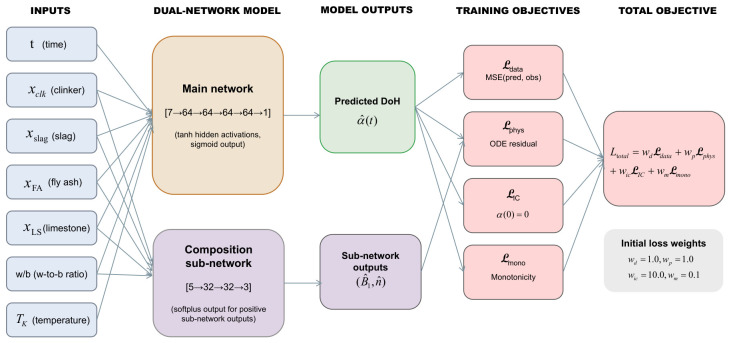
PINN framework architecture for multi-component cement hydration. Main network: 5-layer fully-connected [7→64→64→64→64→1] with tanh activations and sigmoid output. Composition sub-network: [5→32→32→3] with softplus activations predicting the effective kinetic parameters $\left({\hat{B}}_{1},\hat{n}\right)$ that enter the ODE residual. Loss weights (fixed): $w_{d}=1.0$, $w_{p}=1.0$, $w_{\mathrm{ic}}=10.0$, $w_{m}=0.1$.

Figure 1 presents the dual-network PINN framework. Different blend compositions require different effective kinetic parameters $\left({\hat{B}}_{1},\hat{n}\right)$, so one fixed parameter set cannot represent the full slag/fly ash space. A composition sub-network maps the blend vector to these parameters, which enter the Avrami–Erofeev–Arrhenius ODE residual (Equation (4)). This structure separates composition-dependent parameterization from temporal prediction and penalizes kinetic inconsistency at sampled collocation points. The main network maps the 7-dimensional input $x=[t,x_{\mathrm{clk}},x_{\mathrm{slag}},x_{\mathrm{FA}},x_{\mathrm{LS}},w/b,T_{K}]$ to the predicted degree of hydration $\hat{\alpha }$. The composition sub-network provides the effective rate pre-factor ${\hat{B}}_{1}$ and Avrami exponent $\hat{n}$ used in the ODE residual. The full layer dimensions are specified in Section 2.5 and Figure 1. The training objective combines data, ODE-residual, initial-condition, and monotonicity loss terms.

### 2.2. Datasets

Two public isothermal calorimetry datasets were combined into a unified training corpus.

#### 2.2.1. Zenodo Multi-Component Dataset

We used the isothermal calorimetry database of 65 industrially produced cements published by Šmilauer et al. [42] and deposited at Zenodo (record 15212785). The database contains heat-flow and released-heat data from isothermal calorimetry at 20 °C, with water/cement ratios mainly in the 0.40–0.50 range. The cement types span CEMI, CEMII, CEMIII, and CEMV categories, covering a range of clinker (27–97%), slag (0–73%), fly ash (0–24%), and limestone (0–28%) proportions. The Fit-Affinity files provide the degree of hydration α(t) and the fitted parameters $Q_{\mathrm{pot}}$, $B_{1}$, $B_{2}$, η, $\alpha_{\infty }$, and $E_{a}$. We use α(t) as the training target and $Q_{\mathrm{pot}}$ to convert between degree of hydration and cumulative heat. The remaining parameters belong to a different affinity-based kinetic model; they are reported for reference but do not supervise or constrain the network. The cumulative measurement period is up to 9150 h per system, yielding approximately 450 data points per system (*N* = 29,307 total).

#### 2.2.2. MDPI Fly Ash Dataset

To supplement the Zenodo data—which was measured exclusively at 20 °C—with multi-temperature coverage, we manually extracted the isothermal calorimetry data published by Kuryłowicz-Cudowska [43] (MDPI Minerals 12(11):1471) from the tables therein. This dataset comprises 12 systems: three mortar mixtures (OPC, OPC + 10% FA, OPC + 20% FA) at w/b=0.40, each cured at four isothermal temperatures (23, 33, 43, and 53 °C). Each system contained six cumulative heat measurements recorded at 12, 24, 48, 72, 120, and 165 h, yielding a total of 72 data points. The degree of hydration was determined as $\alpha =Q\left(t\right)/Q_{\mathrm{pot}}$, where $Q_{\mathrm{pot}}$ was estimated from the binder composition using $Q_{\mathrm{pot}}=x_{\mathrm{clk}}\times 500+x_{\mathrm{FA}}\times 180$ J/g. In this work, α(t) represents the overall, or bulk, degree of reaction of the binder system and is defined as the ratio of the cumulative heat released at time *t* to the total potential heat $Q_{\mathrm{pot}}$ of the blend. This definition is consistent with standard isothermal calorimetry practice [7], but it should not be interpreted as the clinker-phase degree of hydration. In multi-component systems, α(t) reflects the combined contributions of clinker, slag, and fly ash reactions, weighted according to their respective heat potentials.

#### 2.2.3. Data Split

The full dataset of $N_{\mathrm{sys}}=77$ systems was divided at the system level, rather than the row level, to prevent data leakage: 65% for training (50 systems, 19,022 data points), 15% for validation (11 systems, 4457 points), and 20% for testing (16 systems, 5900 points). The test systems were kept completely separate from the model throughout development. Because the split was performed system-wise, every test system corresponds to a genuinely unseen blend composition and does not benefit from training data drawn from similar formulations.

**Figure 2:**
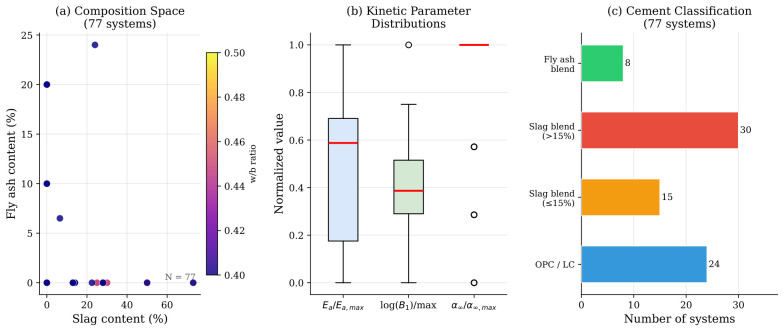
Overview of the unified dataset containing 77 systems. (**a**) Composition space showing slag and fly ash contents, with points colored according to the w/b ratio. (**b**) Normalized distributions of the fitted kinetic parameters ($E_{a}$, $B_{1}$, and $\alpha_{\infty }$). (**c**) Number of systems per cement category: OPC/LC (24), low-slag ≤15% (15), high-slag >15% (30), fly ash blend (8).

Figure 2 summarizes all 77 systems in the unified dataset, including the composition space (slag versus fly ash content), the distributions of the kinetic parameters ($E_{a}$, $B_{1}$, and $\alpha_{\infty }$), and the associated cement classifications.

### 2.3. Input Feature Engineering

The 7-dimensional input vector contains time, binder composition, water-to-binder ratio, and temperature. Processing these variables jointly allows one model to generate composition-conditioned predictions:

(3) $$x=[t,x_{\mathrm{clk}},x_{\mathrm{slag}},x_{\mathrm{FA}},x_{\mathrm{LS}},w/b,T_{K}]\;$$

Each sample is represented by a 7-dimensional input vector, where *t* denotes elapsed time (h), $x_{i}$ represents the mass fraction of each binder component (clinker, slag, fly ash, limestone), w/b is the water-to-binder ratio, and $T_{K}$ is the isothermal curing temperature in kelvin. All input variables are scaled to the range [0,1] using min–max normalization fitted on the training set. The output α∈[0,0.85] denotes the degree of hydration and is left unnormalized because it is already naturally bounded.

### 2.4. Physics Model

The governing equation for isothermal hydration is based on the Avrami–Erofeev kinetic model with Arrhenius-type temperature dependence. This model is selected because it mechanistically captures the three canonical phases of hydration—induction, acceleration, and deceleration—through nucleation-growth theory, and its rate pre-factor $B_{1}$ and nucleation exponent *n* are well-constrained by the isothermal calorimetry data available in our dataset:

(4) $$\frac{d\alpha }{dt}=n\;\cdot \;B_{1}\;\cdot \;\left(1-\alpha \right)\;\cdot \;{\left[-\ln(1-\alpha )\right]}^{(n-1)/n}\;\cdot \;\exp\;\left(-\frac{E_{a}}{R}\left(\frac{1}{T_{K}}-\frac{1}{T_{\mathrm{ref}}}\right)\right)$$

where $B_{1}$ is a rate pre-factor (s^−1^), *n* is a nucleation exponent (dimensionless), $E_{a}$ is the apparent activation energy (J/mol), R=8.314 J/(mol·K) is the gas constant, and $T_{\mathrm{ref}}=293.15$ K (20 °C). The composition-dependent effective parameters ${\hat{B}}_{1}$ and $\hat{n}$ are predicted by a dedicated composition sub-network (Section 2.5). The apparent activation energy $E_{a}$ is held fixed at 40 kJ/mol, a representative value within the literature range (30–45 kJ/mol) and close to the median fitted $E_{a}$ of our dataset (≈38 kJ/mol).

### 2.5. PINN Architecture

Blend composition and hydration time are handled by separate sub-networks. Because different blends follow the same Avrami–Erofeev ODE form but with different kinetic constants, the composition sub-network maps the blend vector to kinetic parameters, while the main network predicts the time-dependent α(t) trajectory. This separation prevents the compositional degrees of freedom from interfering with the ODE learning dynamics and enables the extraction of effective, composition-conditioned parameters as a by-product of training.

The PINN consists of two sub-networks:

Main network: A 5-layer fully-connected network with architecture $R^{7}\to 64\to 64\to 64\to 64\to R^{1}$, using tanh hidden activations and sigmoid output activation to enforce $\hat{\alpha }\in \left(0,1\right)$.

Composition sub-network. A 3-layer network $R^{5}\to 32\to 32\to R^{3}$ (softplus activations) that maps the composition vector $\left(x_{\mathrm{clk}},x_{\mathrm{slag}},x_{\mathrm{FA}},x_{\mathrm{LS}},w/b\right)$ to three outputs. The two effective parameters $\left({\hat{B}}_{1},\hat{n}\right)$ (rate pre-factor and Avrami exponent) enter the ODE residual; the third output is not used in any loss term. Softplus activations ensure positivity of the predicted parameters. All layers use Xavier normal initialization.

### 2.6. Loss Function

The training objective must fit the experimental data and penalize deviations from the governing ODE. It therefore contains four weighted loss terms:

(5) $$L=w_{d}\;L_{\mathrm{data}}+w_{p}\;L_{\mathrm{phys}}+w_{\mathrm{ic}}\;L_{\mathrm{IC}}+w_{m}\;L_{\mathrm{mono}}\;$$

Data loss: $L_{\mathrm{data}}=\frac{1}{N}\sum_{i=1}^{N}{\left({\hat{\alpha }}_{i}-\alpha_{i}\right)}^{2}$.

Physics residual: The physics residual penalizes deviations from Equation (4) at $N_{c}$ collocation points sampled by Latin hypercube sampling in the 7D input space:

(6) $$L_{\mathrm{phys}}=\frac{1}{N_{c}}\sum_{j=1}^{N_{c}}{\left(\frac{1}{\Delta t}\frac{\partial \hat{\alpha }}{\partial t^{*}}{|}_{j}-\mathrm{RHS}\left({\hat{\alpha }}_{j},T_{K,j}\right)\right)}^{2}\;$$

where $t^{*}\in \left[0,1\right]$ is normalized time, Δt is the training time range (h), and $\partial \hat{\alpha }/\partial t^{*}$ is computed via automatic differentiation.

Initial condition: $L_{\mathrm{IC}}=\frac{1}{N_{c}}\sum_{j}\hat{\alpha }{\left(x_{j}{|}_{t^{*}=0}\right)}^{2}$ penalizes deviations from α(t=0)=0.

Monotonicity: $L_{\mathrm{mono}}=\frac{1}{N_{c}}\sum_{j}\max\;{\left(0,\;-\frac{\partial \hat{\alpha }}{\partial t^{*}}\right)}^{2}$ penalizes non-physical decreases in α.

Loss weights were set to $w_{d}=1.0$, $w_{p}=1.0$, $w_{\mathrm{ic}}=10.0$, and $w_{m}=0.1$ and kept fixed throughout training. The initial-condition weight is larger because α(0)=0 is an exact boundary condition for every system. This weight reduces nonzero offsets at the start of the predicted hydration curve. The total number of collocation points was fixed at $N_{c}=3000$. To reduce computational cost, the monotonicity constraint $L_{\mathrm{mono}}$ was evaluated at each Adam step on a subset of 1000 randomly chosen collocation points. Informal hyperparameter sweeps with $N_{c}\in \left\{1500,3000,5000\right\}$ produced negligible differences in held-out performance, confirming that results are insensitive to $N_{c}$ across this range.

We tested the sensitivity to the loss weights with a one-at-a-time analysis around $\left(w_{d},w_{p},w_{\mathrm{ic}},w_{m}\right)=\left(1,1,10,0.1\right)$. Each weight was evaluated at a lower and a higher value, giving nine configurations in total. Test $R^{2}$ ranged from 0.984 to 0.987, and sMAPE ranged from 23.8% to 28.0%. Changing the fixed physics weight $w_{p}$ by a factor of 100 (0.1 to 10) changed $R^{2}$ by less than 0.004, whereas $w_{\mathrm{ic}}$ had the largest effect on accuracy. Removing the monotonicity weight was the only tested change that produced non-monotonic predictions: the violation rate was 0.05% at $w_{m}=0$ and zero for every configuration with $w_{m}>0$. These results support the intended role of $w_{m}$ within the tested configurations.

### 2.7. Training Procedure

Training proceeded in two phases following the established two-stage PINN protocol [44]:

1. Adam phase (3000 epochs, lr = $1\times 10^{-3}$): Global exploration of the loss landscape.
2. L-BFGS phase (500 iterations, strong Wolfe line search): Local refinement to high-precision solution.

A best-modelcheckpoint was maintained throughout training: at each logged epoch, the model state was saved in memory if validation loss improved, and the best checkpoint was restored at the end of training to prevent the L-BFGS optimizer from diverging to degenerate solutions in unlogged iterations. Total training time was 49.4 s on an Intel CPU (no GPU required).

### 2.8. Baseline Models

We compare the PINN against two baselines chosen to evaluate the contribution of the physics constraint: an ablation ANN with identical architecture and training protocol but no physics loss, and a non-parametric random forest that represents the typical data-driven benchmark in the cement ML literature [18,23].

- ANN (ablation): Same architecture as the PINN main network, including sigmoid output activation enforcing $\hat{\alpha }\in \left(0,1\right)$, trained with $L=L_{\mathrm{data}}$ only. Training protocol matches PINN: Adam (3000 epochs, lr = $10^{-3}$) followed by L-BFGS (500 iterations, strong Wolfe line search), with best-checkpoint restoration to prevent L-BFGS divergence. Because the architecture, optimizer, and training data are shared, this comparison evaluates the addition of the physics-based loss terms while recognizing run-to-run training randomness.
- Random Forest (RF): 200 trees with a maximum depth of 15, with all other settings kept at the default values in scikit-learn.

### 2.9. Evaluation Metrics

Model performance is quantified with four metrics computed on the held-out test set. For predictions ${\hat{\alpha }}_{i}$ and measurements $\alpha_{i}$ (i=1,…,N) with sample mean $\bar{\alpha }$,

(7) $$\mathrm{RMSE}=\sqrt{\frac{1}{N}\sum_{i=1}^{N}{\left({\hat{\alpha }}_{i}-\alpha_{i}\right)}^{2}},\;\mathrm{MAE}=\frac{1}{N}\sum_{i=1}^{N}\left|{\hat{\alpha }}_{i}-\alpha_{i}\right|,$$

(8) $$R^{2}=1-\frac{\sum_{i}{\left(\alpha_{i}-{\hat{\alpha }}_{i}\right)}^{2}}{\sum_{i}{\left(\alpha_{i}-\bar{\alpha }\right)}^{2}},\;\mathrm{sMAPE}=\frac{100\%}{N}\sum_{i=1}^{N}\frac{\left|{\hat{\alpha }}_{i}-\alpha_{i}\right|}{(\left|{\hat{\alpha }}_{i}\right|+\left|\alpha_{i}\right|+\epsilon )/2},\;\epsilon =10^{-6}.$$

The symmetric mean absolute percentage error (sMAPE) is reported because it is bounded, and ϵ prevents division by zero when both the measured and predicted values approach zero. This property is useful during early hydration, when ordinary MAPE can become unstable.

## 3. Results

### 3.1. Overall Predictive Performance

Table 1 summarizes the predictive performance of the PINN, ANN, and RF on the held-out test set (16 unseen systems).

Compared with the same-architecture ANN, the PINN lowers sMAPE from 34.9% to 25.3%, a relative reduction of 28%. Only this overall sMAPE difference is statistically significant; the RMSE, MAE, and $R^{2}$ differences are not (Section 3.2). The largest observed stage-resolved sMAPE difference occurs at α<0.1, but no separate significance test was performed for that interval. Because the two neural models share the architecture, optimizer, and training data, their comparison evaluates the addition of the ODE-based loss terms. Run-to-run randomness prevents strict causal attribution from this single fitted pair. The PINN achieves RMSE = 0.038 (relative to $\alpha_{\max}=0.85$, a 4.5% relative error) and has better point estimates than the ANN for all four metrics.

All three models achieve $R^{2}>0.98$ on the full test set, confirming that the seven-dimensional input representation is sufficient to capture the composition- and temperature-dependent variation in hydration kinetics. The $R^{2}$ difference between the PINN (0.9864) and RF (0.9868) is 0.0004 and falls within the random sampling uncertainty of the 16-system test set, so bulk accuracy metrics cannot meaningfully discriminate the two models. RF achieves the lowest absolute errors (RMSE = 0.037, $R^{2}=0.987$) and sMAPE (18.4%) under full training data, reflecting its strength as a non-parametric interpolator within the training distribution (see Section 4 for a detailed analysis).

**Figure 3:**
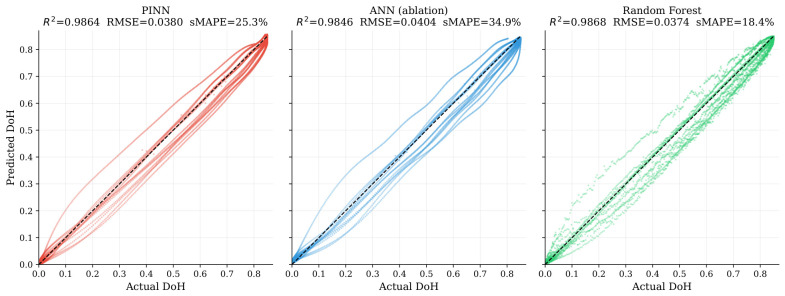
Predicted versus actual degree of hydration ($\hat{\alpha }$ vs. α) for all test systems (16 systems, 5900 points). Each colored curve traces the time-ordered prediction path of one test system; the dashed line is the 1:1 ideal. The PINN (left) achieves tighter agreement in the low-DoH regime (α<0.2) compared to the same-architecture ANN (center), consistent with the sMAPE improvement from 34.9% to 25.3% reported in Table 1. RF (right) achieves comparable or lower absolute error on bulk metrics; the meaningful PINN vs. RF distinction lies in the physical consistency of rate curves rather than aggregate accuracy.

Figure 3 presents predicted versus actual α scatter plots for all three models on the test set. Compared to the same-architecture ANN without physics, the PINN predictions cluster more tightly around the 1:1 line in the low-α regime (<0.2), consistent with the 28% sMAPE improvement reported in Table 1. RF and PINN scatter distributions are visually similar on bulk metrics; the meaningful PINN–RF distinction lies in the physical consistency of the predicted rate curves (Section 3.8).

**Figure 4:**
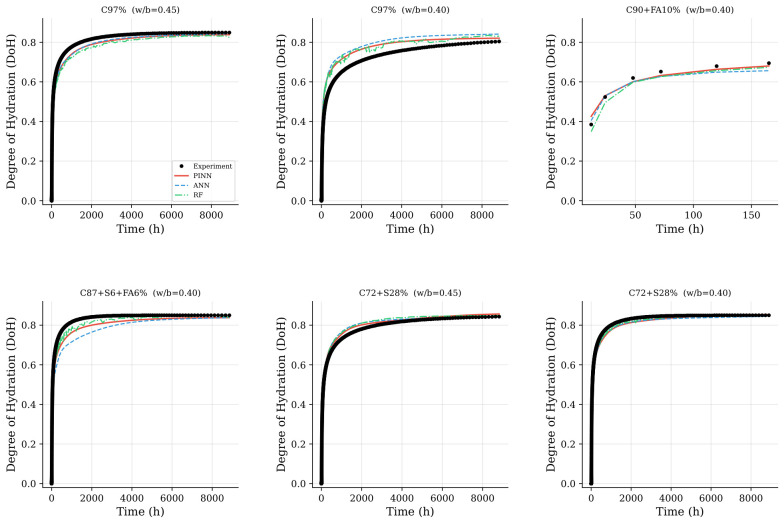
Representative degree-of-hydration curves α(t) are shown for six held-out test systems representing the held-out test composition range: pure OPC at two w/b ratios (top left and center), a fly ash blend with FA10% (top right), a ternary blend C87%+S6%+FA6% (bottom left), and high-slag systems C72%+S28% at w/b=0.45 and 0.40 (bottom center and right). Experimental measurements are shown as filled circles; PINN predictions as solid red lines; ANN predictions as dashed blue lines; and RF predictions as dash–dot green lines. Although all models reproduce the typical sigmoidal shape, the PINN shows smaller deviations than the same-architecture ANN around the induction-to-acceleration transition.

**Figure 5:**
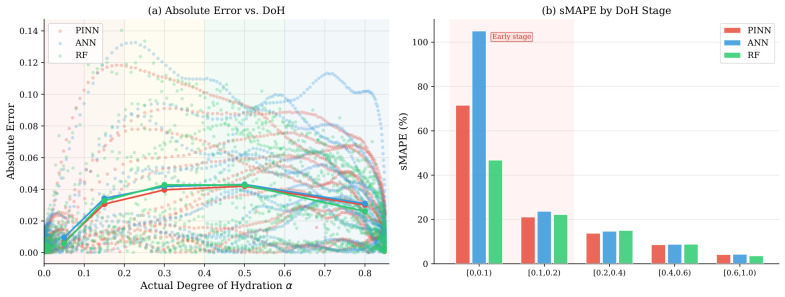
Stage-specific error analysis. (**a**) Absolute error as a function of the actual degree of hydration (DoH) for the PINN (red), ANN (blue), and RF (green), with line segments connecting the mean error within each bin. (**b**) sMAPE across different DoH intervals. The largest observed PINN–ANN difference occurs at α<0.1; no separate significance test was performed for this interval.

Across the six representative test systems in Figure 4, the PINN has smaller deviations than the same-architecture ANN around the induction-to-acceleration transition, where the stage-resolved sMAPE difference is largest. The selected set comprises pure clinker cement (C97%, w/b=0.45), a ternary blend (C87%+S6%+FA6%, w/b=0.40), and high-slag cement (C72%+S28%) at both w/b ratios, thus sampling representative compositions in the held-out test set. All three models reproduce the characteristic sigmoidal behavior. The PINN has lower sMAPE than the same-architecture ANN in the early-time shoulder region (α<0.1), while RF has the lowest pointwise relative error in this interval (Figure 5).

### 3.2. Statistical Significance of Performance Differences

The point estimates in Table 1 differ only marginally for several metrics, so we evaluated their statistical uncertainty rather than comparing the point estimates alone. The 16 held-out systems are the statistically independent experimental units; the 5900 time points are not independent because measurements within each calorimetry curve are strongly autocorrelated. We therefore used a cluster (per-system) bootstrap. The 16 test systems were resampled with replacement B=10,000 times. Each metric and pairwise model difference was recomputed for every resample to obtain percentile 95% confidence intervals (CIs) and two-sided bootstrap *p*-values. These intervals quantify uncertainty arising from test-system sampling, conditional on the fitted models; the complementary run-to-run optimization variability across random seeds is assessed separately in the training-stability and data-efficiency analyses below. Table 2 reports the results.

The cluster bootstrap analysis gives different conclusions for the bulk-accuracy metrics and sMAPE. For the three bulk-accuracy metrics (RMSE, MAE, $R^{2}$), every pairwise difference among the PINN, ANN, and RF has a 95% CI that includes zero (bootstrap p=0.29–0.86); these differences therefore cannot be distinguished from sampling noise and the models are statistically indistinguishable on aggregate accuracy. By contrast, the sMAPE differences are significant in every pairing. The PINN reduces sMAPE relative to the same-architecture ANN by 9.6 points (95% CI [5.2,13.5], p<0.001). RF attains an sMAPE 6.9 points lower than the PINN (95% CI [3.8,10.2], p<0.001). The ANN–RF gap is 16.5 points (95% CI [11.5,20.6], p<0.001). A point-level paired Wilcoxon signed-rank test, included for completeness, agrees in direction with all of these comparisons but—because it treats the 5900 points as independent—inflates significance and is reported here only as a secondary check. We therefore base the interpretation on the cluster bootstrap. The PINN has a statistically significant overall sMAPE advantage over the unconstrained network, and the stage-resolved analysis shows that the largest difference occurs during early hydration. RF remains the most accurate model on pointwise error. No model is significantly better than the others on aggregate RMSE, MAE, or $R^{2}$ within the studied composition range. Relative to RF, the PINN’s demonstrated difference is the explicit physics-based regularization of the predicted hydration-rate curves (Section 3.8).

### 3.3. Stage-Resolved Error Analysis

Figure 5 decomposes test-set errors into five DoH intervals [0,0.1), [0.1,0.2), [0.2,0.4), [0.4,0.6), [0.6,1.0). In the earliest hydration stage (α<0.1), the PINN attains an sMAPE of 71.6%, compared with 105.1% for the same-architecture ANN and 46.8% for RF. The PINN therefore has lower sMAPE than the neural network baseline in the low-DoH regime, although RF has the lowest pointwise relative error in this interval. This stage-specific comparison is descriptive; statistical significance was evaluated for the overall test-set metrics. The large percentage errors in this interval reflect the near-zero denominator: even small absolute deviations produce large relative errors. For α>0.2, all three models have broadly comparable accuracy.

### 3.4. Performance by Supplementary Cementitious Material Type

**Figure 6:**
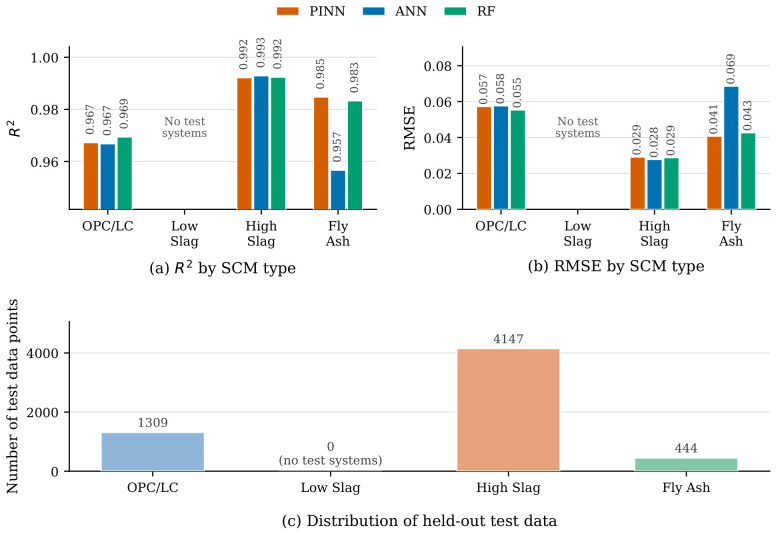
Predictive performance grouped by supplementary cementitious material type. (**a**) $R^{2}$ and (**b**) RMSE for each model across the SCM categories represented in the held-out test split. The low-slag category is shown for completeness but has no held-out test systems in the present split. (**c**) Distribution of held-out test data points in each category. The PINN shows consistent performance across the represented SCM types.

As shown in Figure 6, each model was also assessed separately across supplementary cementitious material categories in the held-out test split. The test split contains OPC/LC, high-slag, and fly ash systems, whereas no low-slag system was assigned to the held-out test set; therefore, low-slag metrics are not reported in this subgroup analysis. All 15 low-slag systems are present in the training and validation data, so the model is fitted in that composition region. We restrict our generalization claims to the categories that were held out (OPC/LC, high-slag, and fly ash) and do not claim validated generalization for the low-slag category. For the represented SCM categories, both neural models have high $R^{2}$ values; ANN errors vary more across the relatively underrepresented fly ash blends. Because the fly ash category contains fewer held-out test data points than the OPC/LC and high-slag categories, this difference should be considered preliminary; a larger dataset with more fly ash systems would be needed to confirm the trend with greater confidence. Even so, within the test split, the PINN performs at least as consistently as the ANN on the underrepresented fly ash category; we treat this as a preliminary in-distribution observation rather than evidence of a general robustness advantage.

### 3.5. Cumulative Heat of Hydration

**Figure 7:**
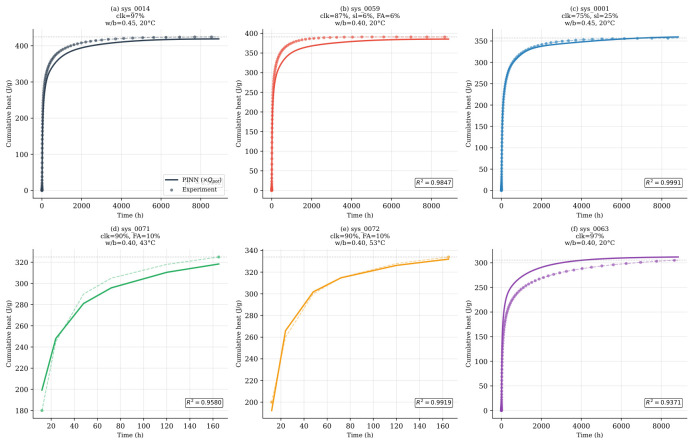
Cumulative heat of hydration, $Q\left(t\right)=\hat{\alpha }\left(t\right)\times Q_{\mathrm{pot}}$, for six representative test systems, shown as PINN predictions (solid lines) against experimental measurements (points). System-level $R^{2}$ values are annotated in each panel. The panels include pure OPC (**a**), a ternary blend (**b**), a high-slag system (**c**), and fly ash systems at different curing temperatures (**d**–**f**).

Mass-concrete thermal analysis uses cumulative heat release, $Q\left(t\right)=\alpha \left(t\right)\;\cdot \;Q_{\mathrm{pot}}$ (J/g), as the required input for structural FEA codes, rather than α(t) itself. Because $Q_{\mathrm{pot}}$ is a known material property available in both datasets, converting degree of hydration to heat release is exact and does not require any additional fitting. Figure 7 shows the predicted Q(t) curves for six representative test systems. The corresponding system-level $R^{2}$ values range from 0.937 to 0.999, indicating that the PINN can provide a reliable surrogate for cumulative heat release in mass-concrete thermal analysis.

### 3.6. Training Convergence

**Figure 8:**
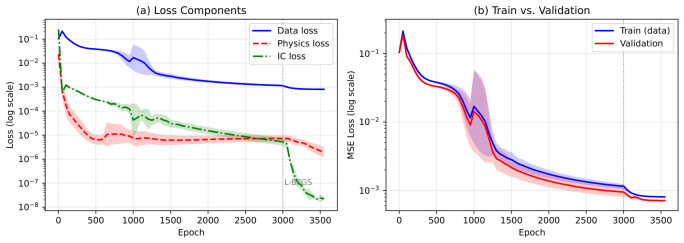
PINN training convergence on a logarithmic scale, shown as the geometric mean over five independent random seeds with a shaded ±1 standard-deviation band computed in log (loss) space. (**a**) Loss components during training: data loss (blue solid line), physics residual $L_{\mathrm{phys}}$ (red dashed line), and initial-condition loss (green dash–dot line). The vertical dotted line indicates the transition from Adam optimization (epochs 0–3000) to L-BFGS refinement. (**b**) Training versus validation MSE loss; their similar final values indicate limited overfitting. The timing of the data-loss drop varies among seeds, producing the wider band near epoch 1000, but the five runs reach similar final losses after the Adam→L-BFGS schedule.

For Figure 8, the two-stage training protocol (Adam followed by L-BFGS) was repeated with five independent random seeds (0, 1, 2, 3, and 42) on the same data split. Per-epoch losses were aligned at the common logged optimization steps. Each curve shows the geometric mean across the five seeds with a ±1 standard-deviation band computed in log-loss space; the band is therefore multiplicative and remains positive. Figure 8 illustrates the resulting training loss history. The physics residual $L_{\mathrm{phys}}$ drops from $2.4\times 10^{-2}$ at epoch 1 to below $10^{-5}$ within the first ∼400 epochs and remains small thereafter. This result indicates close agreement with the Avrami–Erofeev ODE at the sampled collocation points. The data loss decreases sharply around epoch 1200. The timing of this decrease varies among seeds, producing the wider band near epoch 1000, but the five runs reach similar final losses. No evidence of overfitting is found, as the final validation and training losses remain comparable across all seeds (mean final ratio $L_{\mathrm{val}}/L_{\mathrm{train}}=0.89\pm 0.03$).

### 3.7. Data-Efficiency Analysis

**Figure 9:**
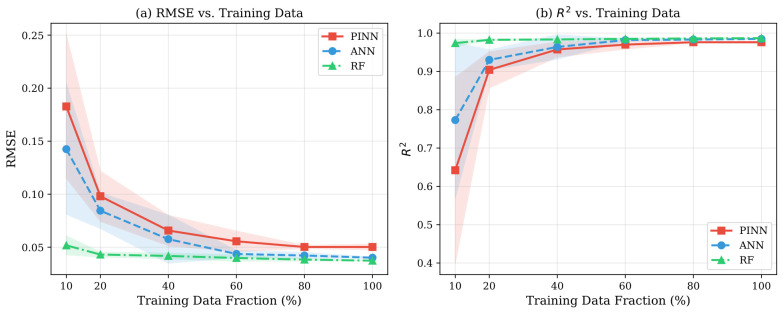
Data-efficiency comparison across training data fractions (10–100% of 50 training systems, i.e., 5–50 systems). (**a**) RMSE and (**b**) $R^{2}$ on the fixed 16-system test set. Shaded bands indicate ±1 standard deviation across eight independently seeded system subsets per fraction. PINN (red squares), ANN (blue circles), and RF (green triangles). At 10% training data (5 systems), both neural models have large and comparable seed-to-seed variation; from 20% onward, all models are more stable. Across every fraction, the ANN mean $R^{2}$ matches or exceeds the PINN’s, so the physics constraint confers no data-efficiency or seed-stability advantage in this sweep.

To quantify model behavior under limited data, we trained the PINN, ANN, and RF on progressively increasing subsets of the training data (10–100% of the 50 training systems). Each fraction was repeated with eight independently seeded system subsets (increased from three to improve the variance estimate) and evaluated on the fixed 16-system test set. Each seed changes both the selected training subset and the model initialization, so the standard deviation reflects variability from subset selection and optimization. Figure 9 presents the results.

The eight-seed results do not support a seed-stability or data-efficiency advantage of the PINN over the same-architecture ANN. At 10% training data (5 systems, ≈2300 data points),

- PINN: $R^{2}=0.64\pm 0.24$, RMSE =0.183±0.068.
- ANN: $R^{2}=0.77\pm 0.20$, RMSE =0.142±0.061.
- RF: $R^{2}=0.97\pm 0.01$, RMSE =0.052±0.009.

With only five training systems, both neural models have large seed-to-seed variation: $\sigma_{\mathrm{PINN}}^{R^{2}}=0.24$ and $\sigma_{\mathrm{ANN}}^{R^{2}}=0.20$. Individual PINN runs range from $R^{2}=0.16$ to 0.94, and ANN runs range from 0.29 to 0.96. These five hydration curves do not provide enough composition coverage for either neural model to perform consistently. We also repeated the 10% case with the longer primary-training schedule (Adam 3000 + L-BFGS 500). The PINN variance remained similar ($R^{2}=0.69\pm 0.23$), indicating that the result was not caused by the shorter training schedule.

RF achieves the highest mean $R^{2}$ at every data fraction (0.97 at 10%; 0.98–0.99 at 40–100%) with minimal variance across seeds (standard deviation of $R^{2}$ below 0.01). This consistency arises from RF’s non-parametric nature: decision-tree splits use all ≈2300 within-system data points directly, without the gradient-descent instabilities that affect neural networks when fewer systems are available. However, RF does not encode the ODE or monotonicity penalties and does not produce composition-conditioned kinetic parameters. Its performance on unseen blend families is evaluated separately in Section 4.

By 20% data (10 systems) both neural models become reliable (PINN $R^{2}=0.90\pm 0.05$, ANN $R^{2}=0.93\pm 0.03$), and from 40% onward all three converge to high accuracy ($R^{2}\geq 0.96$) with $\sigma^{R^{2}}<0.04$. Across every fraction, the ANN mean $R^{2}$ equals or exceeds the PINN’s, and the PINN’s seed variance is not systematically smaller. At 100% training data within this experiment (Adam 2000 + L-BFGS 200, a shorter protocol than the primary benchmark), the ANN achieves $R^{2}=0.985$ and the PINN achieves $R^{2}=0.977$. The slightly lower values relative to Table 1 (PINN 0.9864, ANN 0.9846, Adam 3000 + L-BFGS 500) reflect the reduced training budget used in this data-efficiency sweep and should not be read as a general property of the architectures.

In this data-efficiency analysis, the physics-constrained model is comparable to, but not better than, the unconstrained ANN. Both reach $R^{2}\approx 0.90$ with about ten training systems, and neither shows a consistent seed-stability advantage. The PINN is distinguished by its explicit ODE and monotonicity regularization (Section 4) and its effective, composition-conditioned kinetic parameters, not by lower sensitivity to data scarcity or random initialization.

### 3.8. Physical Consistency of Predicted Hydration Rates

A physics-informed model should be able not only to predict α(t) accurately but also to generate physically consistent hydration rates, $\dot{\alpha }\equiv d\alpha /dt$. To assess this, two quantitative measures were computed over the full test set (16 systems, 5900 points): (i) monotonicity violations, where $\dot{\alpha }<0$ despite hydration being physically irreversible, and (ii) the total variation (TV) of the predicted rate curve, $\mathrm{TV}=\sum_{i}\left|{\dot{\alpha }}_{i+1}-{\dot{\alpha }}_{i}\right|$, which measures smoothness. Table 3 summarizes the results.

**Figure 10:**
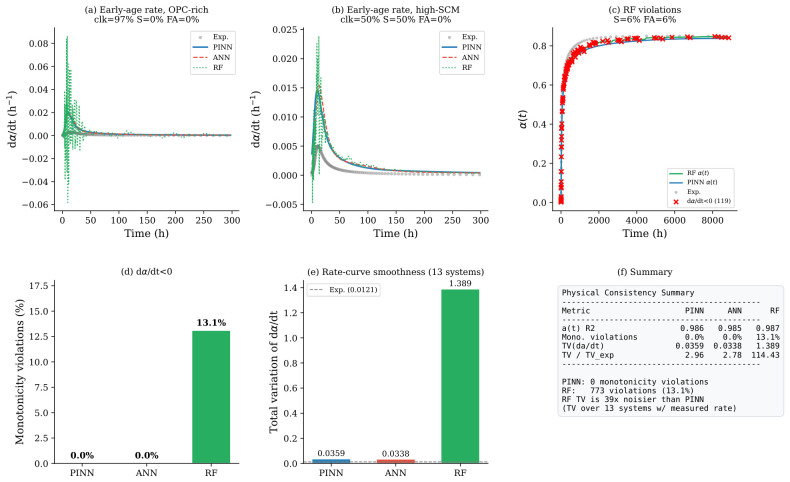
Physical consistency of predicted hydration rates. (**a**) Early-age rate curve dα/dt (t≤300 h) for an OPC-rich test system (97% clinker): PINN and ANN track the measured rate, RF oscillates. (**b**) Early-age rate curve for a high-SCM test system (50% slag): RF exhibits extreme oscillations (±0.04 h^−1^), whereas PINN and ANN remain smooth. (**c**) RF α(t) with monotonicity violations (red crosses: dα/dt<0). (**d**,**e**) Summary bar charts for monotonicity violations and total variation. (**f**) Quantitative summary.

The PINN has no monotonicity violations across the 5900 test points. The ANN, although trained without a monotonicity penalty, also has no violations in this test. By contrast, the RF model produces 773 violations (13.1%), most of which occur in the deceleration regime (α>0.4), where discontinuities between neighboring decision-tree leaf boundaries lead to step-like behavior. Figure 10c shows an example for a representative test system: the RF-predicted α(t) curve oscillates and includes non-physical decreases.

The experimental reference rate is the measured isothermal heat flow normalized by the total heat, ${\dot{\alpha }}_{\exp}=\dot{Q}/Q_{\mathrm{pot}}$; it is not a numerical derivative of α(t). Of the 16 test systems, 13 include measured heat-flow data and are used for the TV comparison. The remaining three MDPI mortar systems report only cumulative heat and are excluded from this comparison. RF has a mean TV approximately 114× the experimental value. The corresponding ratios are 3.0× for the PINN and 2.8× for the ANN. Thus, the PINN and ANN have comparable rate smoothness and are both much smoother than RF. Both neural models also have zero negative-rate violations on the in-range test set.

These results show that the PINN does not improve pointwise accuracy over RF. Its difference from RF is the explicit use of soft ODE-residual and monotonicity penalties during training. The ANN is also monotonic and smooth in this test (zero violations and comparable TV), but it does not include these penalties. RF violates monotonicity on 13.1% of the test points.

## 4. Discussion

### 4.1. Physical Consistency

Unlike the ANN and RF baselines, the PINN is trained with soft ODE and monotonicity penalties. These terms penalize deviations from the Avrami–Erofeev rate equation and negative rates at sampled points in the 7D input space. Within the studied composition range, the trained PINN produces monotonic, non-negative rates on the test set (zero violations; Table 3). This result is empirical rather than a hard guarantee because the constraints are implemented as soft penalties. The leave-one-SCM-out test (Section 4.4) shows that this behavior does not establish accurate prediction for unseen blend families.

**Figure 11:**
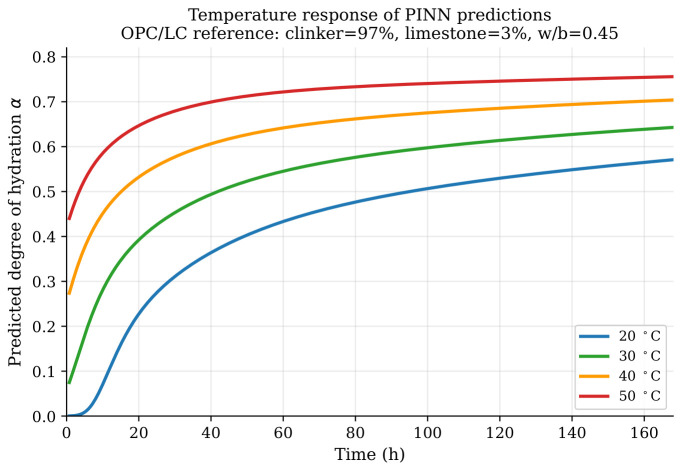
Temperature-response probe of PINN predictions for a fixed OPC/LC reference composition (clinker = 97%, limestone = 3%, w/b=0.45). The curves show predicted degree of hydration at 20, 30, 40, and 50 °C. The comparison is intended to assess qualitative temperature sensitivity rather than to identify a calibrated Arrhenius slope.

The temperature sensitivity of the PINN was analyzed by constructing predictions over the 20–50 °C range for a fixed OPC/LC reference composition (Figure 11). The PINN predicts faster hydration and higher short-term degree of hydration as temperature increases, which is qualitatively consistent with the equivalent age concept and the maturity method standardized in ASTM C1074 [45]. This analysis should be interpreted as a qualitative temperature-response check rather than a calibrated Arrhenius-slope validation, because high-temperature data are limited to a small number of OPC and fly ash systems. The result therefore supports the physical plausibility of the learned temperature trend while also highlighting the need for denser high-temperature data before making quantitative extrapolation claims [33].

**Figure 12:**
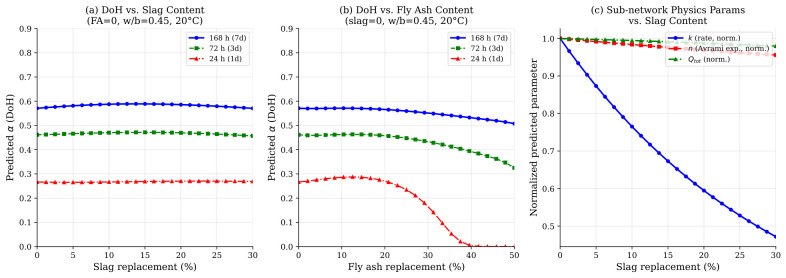
PINN composition sensitivity analysis (all predictions at w/b=0.45, 20 °C). (**a**) Predicted DoH vs. slag replacement at 24, 72, and 168 h; (**b**) predicted DoH vs. fly ash replacement; (**c**) normalized composition sub-network outputs vs. slag content. Fly ash shows strong retarding effect on 1-day DoH; slag effect is mild over the probed range (0–30%).

Composition sensitivity analysis (Figure 12) shows that the PINN predicts the experimentally established trend that fly ash replacement significantly retards early hydration (24 h), consistent with the low pozzolanic reactivity index of Class F fly ash [1,46]. Over the slag range probed here (0–30%), slag replacement has a comparatively minor effect on 7-day DoH, consistent with the hydraulic nature of GGBFS at normal curing temperatures [16,47]. The composition sub-network predicts a rate pre-factor, ${\hat{B}}_{1}$, that declines monotonically as slag content increases, qualitatively matching the well-established dilution effect of slag on clinker hydration rate reported by Lothenbach et al. [7].

The cumulative heat of hydration $Q\left(t\right)=\alpha \left(t\right)\;\cdot \;Q_{\mathrm{pot}}$ predicted by the PINN agrees closely with experimental isothermal calorimetry (Figure 7), with system-level $R^{2}$ values of 0.937–0.999 across the six representative test systems. The ability to predict Q(t) with this accuracy is directly relevant to the thermal analysis of mass-concrete structures using the heat generation rate as input to structural FEA codes [13,14].

### 4.2. Comparison with Prior PINN Studies

The closest prior PINN works in the cement domain are PINN-CHK [40] and EcoBlendNet [41]. PINN-CHK applied PINN to isothermal hydration of plain OPC pastes (3–5 systems), demonstrating the viability of embedding the Avrami–Erofeev ODE but without extending to multi-component blends. EcoBlendNet embedded PDE-based physics constraints for three specific blend formulations (OPC, 45% fly ash, 80% slag) and achieved low approximation error with only 5% training data, demonstrating the data efficiency of physics constraints. Our framework differs from these studies in four ways. First, a dedicated sub-network treats composition as a continuous input, so one model covers the studied slag/fly ash space. Second, temperature is an explicit seventh input rather than a system-level parameter. Third, the dataset contains 77 systems, compared with 3–5 systems in the earlier studies, allowing for evaluation across training-data fractions and compositions. Fourth, the comparison of PINN, ANN, and RF includes both accuracy and physical-consistency metrics. The resulting model is composition-conditioned and outputs effective kinetic parameters.

Relative to the purely data-driven approaches of Lapeyre et al. [18] and Cook et al. [19], which achieved high interpolation accuracy using RF on 235–300 blended systems, the PINN has slightly higher pointwise error but includes explicit ODE-based regularization and outputs effective kinetic parameters. The standard RF formulation does not include these features. The deep forest approach of Han et al. [20], which incorporated topological constraint theory to reduce data requirements for fly ash binders, shares our motivation of embedding domain knowledge; however, their constraints operate at the feature-engineering level rather than as an ODE residual in the loss function, and their model does not include an explicit trajectory-level ODE or monotonicity penalty.

### 4.3. Comparative Analysis: PINN Versus Random Forest

The PINN is not uniformly superior to the ANN or RF. Model selection depends on whether the primary criterion is pointwise accuracy, explicit physical regularization, or performance with limited training data. With the full training set, RF has the best point estimates (RMSE = 0.037, $R^{2}$ = 0.987, and sMAPE = 18.4%). Only the sMAPE differences are statistically significant; the RMSE, MAE, and $R^{2}$ differences are not (Section 3.2). This result is expected: RF is a non-parametric interpolator that excels when test systems lie within the convex hull of training compositions, as is largely the case here given the system-wise 65%/15%/20% training/validation/test split. Two differences are relevant when explicit physical regularization or kinetic parameters are required:

1. No explicit physical constraints: RF predictions are not trained against the Avrami–Erofeev ODE and produce non-monotonic α(t) curves on 13.1% of test points (Table 3), with rate-curve total variation approximately 114× the experimental value.
2. Composition-conditioned effective kinetic parameters: The composition sub-network of the PINN predicts effective kinetic parameters (${\hat{B}}_{1}$, $\hat{n}$) (with the apparent activation energy held fixed rather than fitted) as a function of blend composition. These vary smoothly with composition—for example ${\hat{B}}_{1}$ decreases monotonically with slag content, qualitatively consistent with the dilution effect—although they are effective fit parameters rather than independently validated material constants.

The PINN does not have an absolute-accuracy advantage over RF. Its distinguishing features are the explicit ODE and monotonicity penalties and the composition-conditioned effective parameters.

### 4.4. Limitations

The source data are not evenly distributed across composition or temperature. Of the 77 systems, only seven contain less than 70% clinker, leaving high-replacement blends sparsely represented. In the leave-one-SCM-out tests, mean PINN $R^{2}$ fell below zero for both fly ash (−0.23±0.38) and high slag (−0.60±0.33). The ANN also degraded (0.71±0.06 and 0.89±0.09), whereas RF retained $R^{2}=0.975$ and 0.980 (SD <0.001). These results do not show an extrapolation benefit from the soft ODE and monotonicity penalties; the conclusions drawn here therefore apply only to the composition range represented in the training data. Temperature coverage is similarly narrow: only 72 of the 29,379 calorimetry points vary with temperature, while the remaining ≈99.8% were measured at 20 °C. Accordingly, Figure 11 is used to indicate qualitative trends rather than validated temperature generalization.

**Figure 13:**
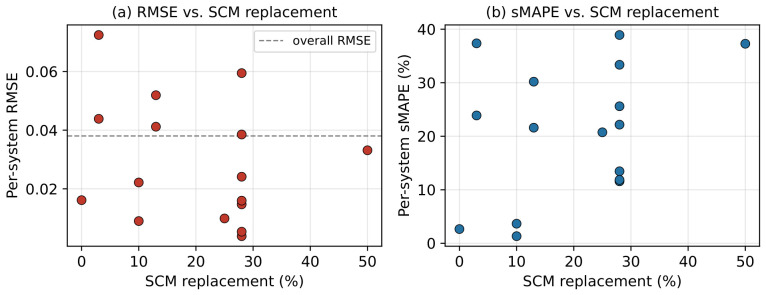
Per-system test error versus SCM replacement level (slag + fly ash + limestone). Each point represents one of the 16 held-out systems; the dashed line marks the overall RMSE. No systematic RMSE trend is observed up to 50% replacement, although the single 50% system has one of the largest sMAPE values.

Interpretation is also limited by the model formulation. The composition sub-network predicts the effective parameters ${\hat{B}}_{1}$ and $\hat{n}$, and the apparent activation energy $E_{a}$ is fixed rather than learned. Their weak correlation with the fitted Fit-Affinity parameters indicates that they are effective fitting quantities, not independently validated material constants. Likewise, the single Avrami–Erofeev ODE represents aggregate heat release and does not resolve the individual reactions of clinker, slag, and fly ash [7]. The available held-out systems span replacement levels up to 50%, and their RMSE is not systematically related to replacement level (Pearson r=−0.25; Figure 13). Nevertheless, only one held-out system has 50% replacement, and its sMAPE is among the largest (≈37%). The current data therefore do not support conclusions for higher replacement levels. Resolving the individual reactions would require phase-resolved measurements or additional kinetic data across temperatures.

Finally, the monotonicity loss $L_{\mathrm{mono}}$ is evaluated at sampled collocation points and cannot guarantee monotonicity between them; a neural-ODE formulation could impose this property structurally [48]. The PINN–ANN comparison also tests the complete physics-informed loss without isolating $L_{\mathrm{phys}}$, $L_{\mathrm{IC}}$, and $L_{\mathrm{mono}}$. A component-wise ablation would be needed to determine how much each term contributes.

## 5. Conclusions

Embedding the Avrami–Erofeev–Arrhenius ODE in the training loss enables one neural network to model the studied blend-composition space without per-blend calibration. The physics-based loss does not improve aggregate accuracy over RF or produce a significant $R^{2}$ gain over the same-architecture ANN. Instead, it explicitly penalizes ODE-residual and monotonicity violations. The model was evaluated on 77 systems spanning 0–73% SCM replacement (clinker 27–100%). The main findings are:

1. The PINN achieves $R^{2}=0.9864$ and sMAPE = 25.3% on unseen test systems; relative to the same-architecture ANN without physics ($R^{2}=0.9846$, sMAPE =34.9%), the overall sMAPE difference is statistically significant, whereas the $R^{2}$ difference lies within sampling uncertainty (Section 3.2). Stage-resolved analysis shows that the largest sMAPE difference occurs during early hydration. Although RF attains lower pointwise error, it does not preserve physically consistent hydration-rate curves.
2. Across eight random seeds, the physics constraint does not provide a data-efficiency or seed-stability advantage over the same-architecture ANN. Both reach $R^{2}\approx 0.90$ with about ten training systems and have large variation when trained on only five systems. The PINN is distinguished by its explicit physics-based regularization and effective, composition-conditioned parameterization, not by reduced variance.
3. The PINN produces zero monotonicity violations (dα/dt<0) across all 5900 test predictions, whereas the RF violates physical monotonicity on 13.1% of points, with rate-curve TV approximately 114× the experimental value.
4. Training completes in under 50 s on a standard CPU, making the framework practical for rapid mix-design iteration.

The trained PINN may serve as a rapid, physics-regularized surrogate for screening blend compositions before experimental testing. The current scope is limited to isothermal conditions and is dominated by high-clinker compositions. Although the data span 27–100% clinker, only 7 of 77 systems fall below 70% (see Section 4.4). Denser low-clinker data and non-isothermal curing histories are needed in future studies. Generalization was evaluated on held-out OPC/LC, high-slag, and fly ash systems. Low-slag systems were included in training and validation but were not independently tested.

Future work could examine 3D-printed concrete with variable temperature histories [49], multiphase kinetic models for high-SCM systems [50,51], and integration with thermodynamic simulators for strength and durability prediction [7,11]. The surrogate modeling approach could also be extended to coupled thermo-mechanical analysis of early-age concrete structures [13,51].

## Figures and Tables

**Table 1:** Predictive performance on the held-out test set (16 systems, 5900 points). sMAPE = symmetric mean absolute percentage error. The arrows in the column headers indicate the optimization direction: ↓ marks metrics for which lower values are better (RMSE, MAE, sMAPE) and ↑ marks metrics for which higher values are better ($R^{2}$). Bold: best per metric.

Model	RMSE ↓	MAE ↓	$R^{2}$ ↑	sMAPE % (↓)
PINN	0.0380	0.0263	0.9864	25.3
ANN	0.0404	0.0280	0.9846	34.9
RF	**0.0374**	**0.0248**	**0.9868**	**18.4**

**Table 2:** Cluster (per-system) bootstrap analysis of the test-set metrics (B=10,000 resamples of the 16 test systems). Values are the point estimate with the 95% confidence interval in brackets; the Δ rows give every pairwise between-model difference with its 95% CI, and the lower block lists the corresponding two-sided bootstrap *p*-values. A CI that contains zero (equivalently p>0.05) indicates a difference that is not statistically significant at the 5% level.

	RMSE ↓	MAE ↓	$R^{2}$ ↑	sMAPE % ↓
PINN	0.038 [0.026, 0.048]	0.026 [0.017, 0.036]	0.986 [0.978, 0.994]	25.3 [20.2, 30.4]
ANN	0.040 [0.026, 0.052]	0.028 [0.018, 0.039]	0.985 [0.974, 0.994]	34.9 [29.8, 39.7]
RF	0.037 [0.025, 0.048]	0.025 [0.016, 0.034]	0.987 [0.978, 0.994]	18.4 [13.3, 23.4]
Δ (PINN−RF)	+0.001 [−0.006, +0.007]	+0.001 [−0.003, +0.007]	−0.000 [−0.005, +0.004]	+6.9 [+3.8, +10.2]
Δ (PINN−ANN)	−0.002 [−0.009, +0.004]	−0.002 [−0.006, +0.002]	+0.002 [−0.002, +0.007]	−9.6 [−13.5, −5.2]
Δ (ANN−RF)	+0.003 [−0.006, +0.010]	+0.003 [−0.003, +0.009]	−0.002 [−0.008, +0.004]	+16.5 [+11.5, +20.6]
*p* (PINN−RF)	0.86	0.59	0.86	<0.001
*p* (PINN−ANN)	0.51	0.43	0.51	<0.001
*p* (ANN−RF)	0.46	0.29	0.46	<0.001

**Table 3:** Physical consistency metrics on the test set. TV = total variation of dα/dt (mean per system); ${\mathrm{TV}}_{\mathrm{ratio}}$ = TV normalized by experimental TV. Monotonicity violations are over all 16 test systems; TV and ${\mathrm{TV}}_{\mathrm{ratio}}$ are computed on the 13 of 16 test systems that carry measured heat-flow data.

Model	Mono. Violations ↓	TV (dα/dt) ↓	TV_ratio_ ↓
Experiment	—	0.0121	1.0×
PINN	0/5900 (0.0%)	0.0359	3.0×
ANN	0/5900 (0.0%)	0.0338	2.8×
RF	773/5900 (13.1%)	1.389	114×

## Data Availability

The primary dataset used in this study is the isothermal calorimetry database of 65 industrially produced cements associated with [42]; it is publicly available at Zenodo: https://doi.org/10.5281/zenodo.15212785. The fly ash supplement data are reproduced from the open-access literature [43]. All model training code and processed data are available from the corresponding author upon reasonable request.

## Abbreviations

The following abbreviations are used in this manuscript:

Adam	Adaptive Moment Estimation (optimizer)
ANN	Artificial Neural Network
CI	Confidence Interval
CPU	Central Processing Unit
DoH	Degree of Hydration
FA	Fly Ash
FEA	Finite Element Analysis
GGBFS	Ground-Granulated Blast-Furnace Slag
GPU	Graphics Processing Unit
IC	Initial Condition
L-BFGS	Limited-memory Broyden–Fletcher–Goldfarb–Shanno (optimizer)
LC	Limestone Cement (limestone-blended Portland cement)
LS	Limestone (component mass fraction)
MAE	Mean Absolute Error
MAPE	Mean Absolute Percentage Error
ML	Machine Learning
MSE	Mean Square Error
ODE	Ordinary Differential Equation
OPC	Ordinary Portland Cement
PINN	Physics-Informed Neural Network
RF	Random Forest
RMSE	Root Mean Square Error
SCM	Supplementary Cementitious Material
sMAPE	Symmetric Mean Absolute Percentage Error
TV	Total Variation
*w*/*b*	Water-to-Binder ratio
